# Control of Pem protein level by localized maternal factors for transcriptional regulation in the germline of the ascidian, *Halocynthia roretzi*

**DOI:** 10.1371/journal.pone.0196500

**Published:** 2018-04-30

**Authors:** Kaori Miyaoku, Ayaki Nakamoto, Hiroki Nishida, Gaku Kumano

**Affiliations:** 1 Asamushi Research Center for Marine Biology, Graduate School of Life Sciences, Tohoku University, Asamushi, Aomori, Japan; 2 Department of Biological Sciences, Graduate School of Science, Osaka University, Machikaneyama-cho, Toyonaka, Osaka, Japan; University of Minnesota Medical Center, UNITED STATES

## Abstract

Localized maternal mRNAs play important roles in embryogenesis, e.g. the establishment of embryonic axes and the developmental cell fate specification, in various animal species. In ascidians, a group of maternal mRNAs, called *postplasmic/PEM* RNAs, is localized to a subcellular structure, called the Centrosome-Attracting Body (CAB), which contains the ascidian germ plasm, and is inherited by the germline cells during embryogenesis. Posterior end mark (Pem), a *postplasmic/PEM* RNAs member, represses somatic gene expression in the germline during cleavage stages by inhibition of RNA polymerase II activity. However, the functions of other *postplasmic/ PEM* RNAs members in germline formation are largely unknown. In this study, we analyzed the functions of two *postplasmic/PEM* RNAs, *Popk-1* and *Zf-1*, in transcriptional regulation in the germline cells. We show that Popk-1 contributes to transcriptional quiescence by controlling the size of the CAB and amount of Pem protein translated at the CAB. Our studies also indicated that zygotic expression of a germline gene starts around the onset of gastrulation and that the decrease of Pem protein is necessary and sufficient for the zygotic germline gene expression. Finally, further studies showed that the decrease of the Pem protein level is facilitated by Zf-1. Taken together, we propose that *postplasmic/PEM* RNAs such as Popk-1 and Zf-1 control the protein level of the transcriptional repressor Pem and regulate its transcriptional state in the ascidian germline.

## Introduction

Germline is a specialized cellular lineage that transmits genetic information to the next generation. In various animals, the germline is set aside from the somatic linage throughout their life cycles. This separation plays a pivotal role in the retention of the unique characteristics of germ cells such as their totipotency and immortality and in protection from being compromised by somatic programs. One strategy for this germline segregation is known as transcriptional repression for somatic genes in the lineage [1– 4]. Owing to the importance of the establishment and maintenance of the germline, regulation of gene expression in the germline has been a key subject of much research over the years [1– 4].

Transcriptional regulation in the germline begins during the early stages of development. In particular, it has been observed in many animals such as the fly *Drosophila melanogaster* [5], the roundworm *Caenorhabditis elegans* [5], the frog *Xenopus laevis* [6], the ascidians *Ciona robusta* (formerly *Ciona intestinalis* type A) [7] and *Halocynthia roretzi* [8] and the sea urchin *Strongylocentrotus purpuratus* [9], in which transcription is globally repressed in the germline cells for certain periods of the embryonic stages when zygotic gene expression has begun in the somatic lineage. This early transcriptional quiescence involves maternal factors such as Polar granule component (Pgc), Germ cell-less (Gcl) and Nanos of *D*. *melanogaster* [10–15], OMA-1/-2 and PIE-1 of *C*. *elegans* [16–18], Nanos1 of *X*. *laevis* [19] and Pem of *C*. *robusta* [7] and *H*. *roretzi* [8]. Most of these maternal factors are localized to a distinct cytoplasmic compartment called the germ plasm together with other maternal factors essential for germline development. They are inherited by germline cells during cell cleavages. At later stages, following the disappearance of these maternal factors, which gives permissive states for transcription, it is suggested in some species that chromatin-based silencing mechanisms could contribute to maintain repression of somatic gene expression while zygotic germline-specific gene expression begins for germ cell formation [2, 3].

**Fig 1:**
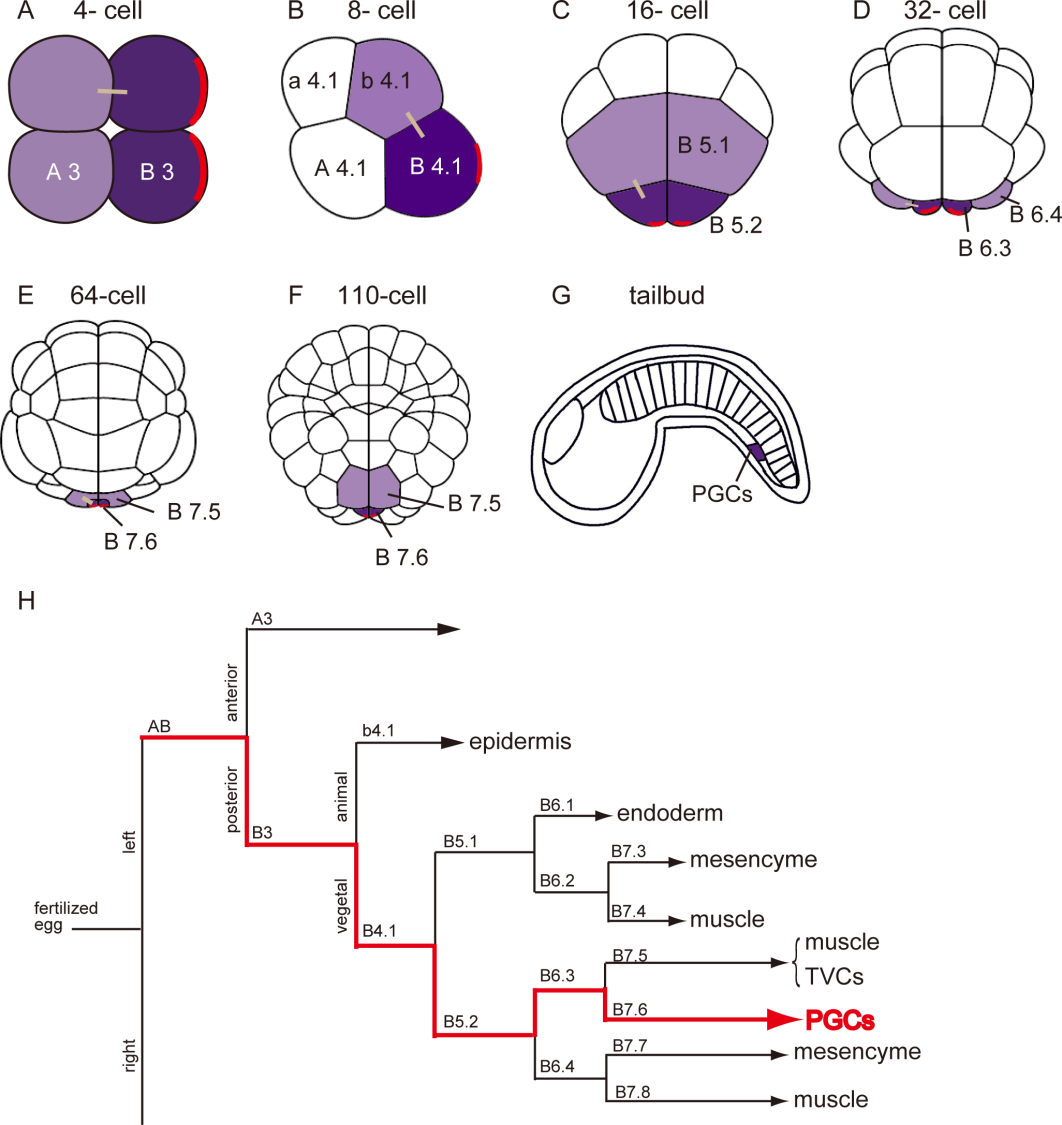
The germline lineage of the *Halocynthia* embryo. (A-G) Schematic diagram of ascidian embryogenesis. 8-cell stage (B) and tailbud stage (G) embryos are viewed laterally. (C-F) are vegetal views. (A), (B) and (G) are oriented with anterior to the left and posterior to the right. (C-F) are oriented with anterior to the top and posterior to the bottom. Germline cells at the cleavage stages (A-F, dark purple) are present at the posterior end of the embryos and divide to produce a somatic daughter (light purple) and a germline daughter at cell divisions. The germline daughters inherit the ascidian germ plasm CAB (A-F, red lines). *postplasmic/PEM* RNAs are localized to the CAB. Two cells connected by short bars are sister cells. During gastrulation germline, cells migrate internally and end up being located near the tip of the tail at the tailbud stage (G). (H) Cell linage during ascidian embryogenesis. The red line indicates germline lineage. Only the B lineage (posterior vegetal region) is shown in detail. PGCs: primordial germ cells, TVCs: trunk ventral cells.

Ascidians belong to the sister group of vertebrates and share chordate characteristics such as notochord and dorsal nervous systems at their larval stage [20–22]. The germline of the ascidian in early development is composed of the posterior-most cells at each cleavage stages (B3 at the 4-cell, B4.1 at the 8-cell, B5.2 at the 16-cell, B6.3 at the 32-cell and B7.6 blastomere at the 64- and 110-cell stages, Fig 1) [23–29]. These cells inherit a sub-cellular structure called the Centrosome-Attracting Body (CAB, Fig 1), which contains electron dense matrix morphologically resembling the germ plasm found in other animals such as *D*. *melanogaster* and *X*. *laevis* [24, 30] and is associated with maternal mRNAs known as *postplasmic/PEM* RNAs [29, 31, 32]. Although 39 and 14 of such localized maternal factors have been identified in *Ciona* and *Halocynthia* embryos, respectively [29, 31, 32], only a few have been investigated for their function in germline development. The most studied is *Posterior end mark* (*Pem*), which was the first *postplasmic/PEM* RNAs member to be identified as a maternally localized mRNA [33] and has recently been shown to repress somatic gene expression in the germline of *C*. *robusta* and *H*. *roretzi* [7, 8]. In the latter species, Pem is further shown to bind to a protein complex called p-TEFb, which phosphorylates Ser2 in the C-terminal domain of RNA polymerase II for transcriptional elongation, and to inhibit its activity [8]. This p-TEFb /RNA-polymerase II-dependent transcriptional repression is also observed in other animals and regulated by Pgc of *D*. *melanogaster* and PIE-1 of *C*. *elegans* [14, 17, 34]. Interestingly, Pem, Pgc and PIE-1 share no sequence similarity and thus independently evolved [8, 35, 36]. Nanos1 of *X*. *laevis* has also been shown to regulate the Ser2 phosphorylation, although molecular mechanism is unknown [19].

In addition to Pem, it is speculated that Popk-1 is the only member of *postplasmic/PEM* RNAs involved in germline formation since it controls the size of the CAB, ascidian version of the germ plasm [37]. In this study, we investigated the roles of the *postplasmic/PEM* RNAs in germline development and show that 1) Popk-1 contributes to repress germline transcription indirectly via regulating proper CAB formation where *Pem* mRNA is localized, 2) decreasing the Pem protein level during embryogenesis is necessary and sufficient for the onset of a zygotic germline gene expression at the gastrula stage, and that 3) Zf-1 mediates downregulation of the Pem protein level.

## Materials and methods

### Animals and embryos

Farmed *Halocynthia roretzi* adults were purchased from local fishermen with the help of Nonai Branch of Aomori City Fisheries Cooperative Association (40°51’3”N 140°48’59”E), Noheji Fisheries Cooperative Association (40°52’21”N 141°7’10”E) and Aomori City Fisheries Promotion Center (40°54’5”N 140°40’29”E) in Mutsu Bay (Aomori, Japan) and of Otsuchi International Coastal Research Center, University of Tokyo (39°21’6”N 141°56’4”E) (Iwate, Japan). No specific permissions were required. The adults were kept under constant light at 8°C to suppress spawning. To induce spawning, the adults were put under dark condition for several hours and then exposed to light in seawater at 11~13°C. Spawned eggs were fertilized with a suspension of non-self-sperm. Embryos were cultured in Millipore-filtered sea water (MFSW) containing 50 mg/L streptomycin sulfate (Sigma) and 50 mg/L kanamycin sulfate (Wako) at 9–13°C.

### Whole-mount in situ hybridization (WISH)

The expression of *FoxD*.*a* (Harore.CG.MTP2014.S128.g04021 in the Aniseed database, https://www.aniseed.cnrs.fr/) [38], Pem (Harore.CG.MTP2014.S480.g14149) and *ADP/ATP translocase* (Harore.CG.MTP2014.S737.g07016) [39] was detected by WISH. WISH was performed as described [40]. The antisense digoxigenin (DIG) -labeled RNA probes for *ADP/ATP translocase* were synthesized using the FE107G06 plasmid clone obtained from the EST database MAGEST [41; http://magest.hgc.jp/] as a template. Stained samples were mounted in 80% glycerol in PBS or VECTASHIELD Mounting Medium with DAPI (Vector Laboratories) and observed with a stereoscopic microscope SZX16 (Olympus) or microscope BX51 (Olympus).

### Actinomycin D treatment

To inhibit transcription, embryos were cultured in MFSW containing 40 μg/mL of actinomycin D (Sigma). The actinomycin D-treated embryos were fixed with 4% paraformaldehyde in fixation buffer (0.5 M NaCl, 0.1 M MOPS, pH 7.5) when DMSO-treated control embryos reached the late neurula stage. Actinomycin D at a concentration of 20 μg/mL has been reported to suppress 70% incorporation with the unincorporated 30% being low-molecular weight RNA in the ascidian *Phallusia nigra* embryos [42]. This concentration also inhibited gene expression in *Halocynthia* embryos [43–45].

### mRNA synthesis, morpholino antisense oligo nucleotide (MO) and microinjection

mRNAs for *Pem*, *Zf-1* and *LacZ* were *in vitro* synthesized with the mMessage mMachine kit (Ambion) and the Poly(A) Tailing kit (Ambion). The plasmids *Pem* in pBluescript-HTB(N) [38] and *LacZ* in pSP6nuβGal were used as templates for *in vitro* RNA synthesis. A template plasmid for *Zf-1* mRNA synthesis was prepared by inserting a PCR-amplifying fragment that contains the entire ORF and 1652 bp of the 3’UTR region of the *Zf-1* gene [46] into pBluescript-HTB(N).

MOs (Gene Tool) to suppress the translation of *Popk-1* (Harore.CG.MTP2014.S69.g03049) [37] and *Pem* [47] were used as described previously. The nucleotide sequence of MO to knockdown Zf-1 (Harore.CG.MTP2014.S839.g14521) was 5’-GCAAGGAATAACAAAAAAGCAGAGA-3’, which covers -25 to -1bp from the starting ATG of the *Zf-1* gene [46]. Standard MO (Gene Tool) was used as a control.

Either fertilized eggs at 45 minutes to 2 hours after fertilization or the germline cells (B 5.2 blastomeres) of the 16-cell stage embryos were microinjected as described previously [48]. The injected amount was one fourth to one fifth of the diameter of the cells (about one hundredth of the volume). For the 16-cell stage microinjection, we confirmed by labeling the injected cells that the descendant cells were not ablated by physical damage from injection. Results from at least three independent experiments were combined for all the data presented in this study.

### Pem protein immunostaining

Detection of Pem protein with the antibody was performed as described [8], with the following exceptions; the concentration of the primary antibody was used at 1/450, 1/750 or 1/1350 instead of 1/25, PBS containing 0.1% Triton X-100 instead of 0.05% was used as a washing solution, and samples were washed with PBS containing 1% Triton X-100 after NH_4_Cl treatment. Samples were mounted in VECTASHIELD Mounting Medium with DAPI (Vector Laboratories) and observed using a microscope BX51 (Olympus) or confocal microscope LSM5 PASCAL (ZEISS). To compare the signals of Pem protein in the germline nucleus, we observed the germline nucleus with confocal microscope and made a Z-series through the nucleus. SYTOX green (Invitrogen) was used as a nuclear marker. A single z-section, where nuclear Pem protein signal was strongest in the Z-series, was selected. This observation was undertaken in the germline nucleus on both sides of the embryo. This procedure allowed us to compare the level of strongest Pem protein signal in germ line nuclei between the control embryos and experimental embryos.

## Results

### Popk-1 involves germline transcriptional repression

**Fig 2:**
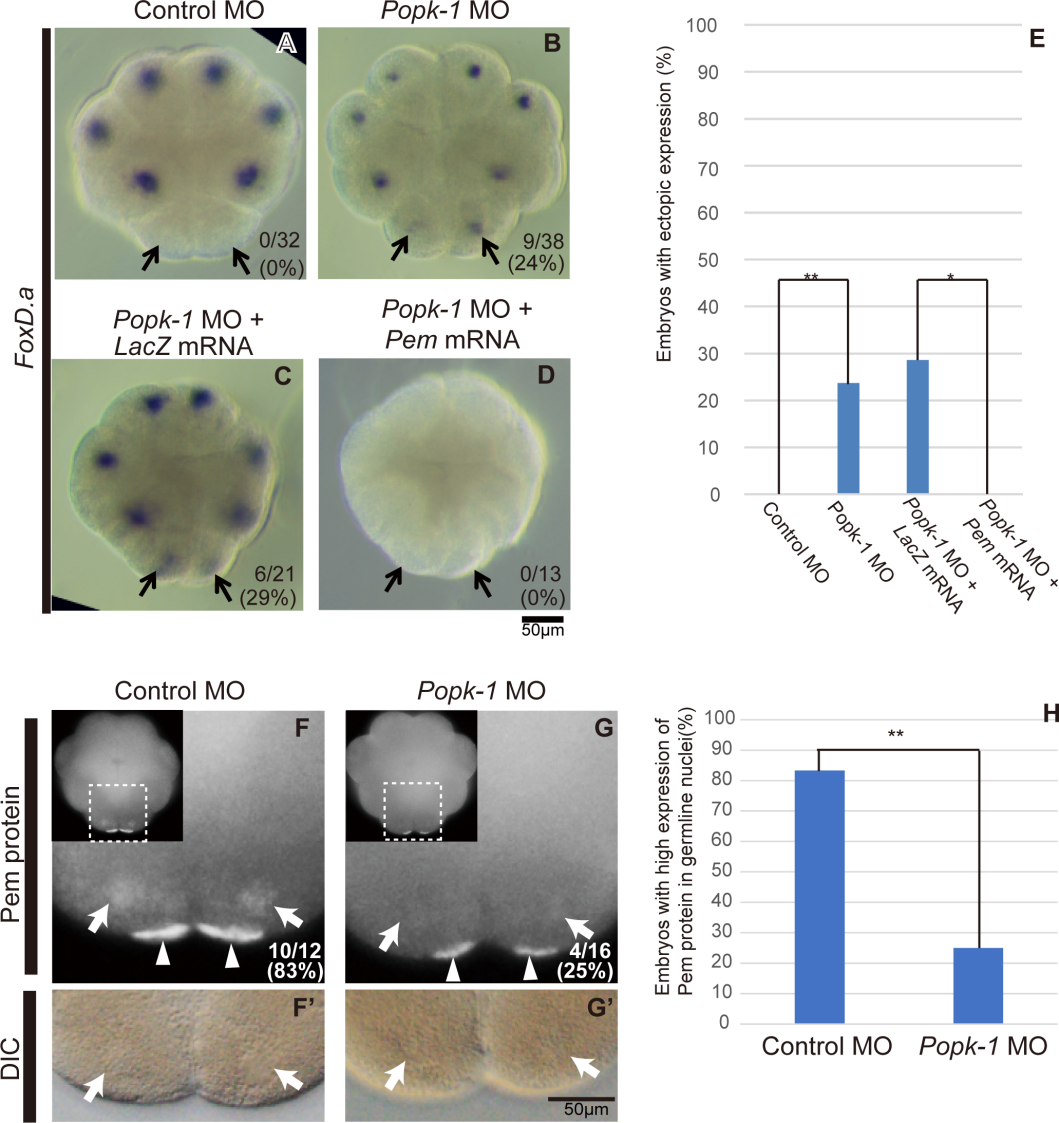
Regulation of germline transcriptional repression by Popk-1. (A-D) Images of 16-cell stage embryos stained by WISH for *FoxD*.*a*. Shown are (A) 1000 pg of control MO-, (B) 1000 pg of *Popk-1* MO-, (C) 1000 pg of *Popk-1* MO and 50 pg of *LacZ* mRNA- and (D) 1000 pg of *Popk-1* MO and 50 pg of *Pem* mRNA-injected embryos. Vegetal views. Anterior is to the top. Arrows indicate germline B5.2 cells. Scale bar, 50 μm. The digits in the bottom right corner indicate the proportions of embryos with ectopic *FoxD*.*a* expression in the germline. The proportions are also shown in E. ** P<0.01, * P<0.05 (one-sided Fisher’s exact test). Although the numbers with the ectopic expression were low in B (24%) and in C (29%), 95% of embryos (n = 19) showed ectopic expression by injection of an increased amount of MO (1500 pg, S1 Fig). (F, G) Fluorescent images of the 16-cell stage embryos stained with anti-Pem antibody. (F) 1000 pg of control MO- and (G) 1000 pg of *Popk-1* MO-injected embryos. White arrows indicate the nuclear signals, and white arrowheads the CAB signals. Pem protein has been shown previously to be present both in the nucleus and CAB in the ascidian germline [8]. Smaller panels in the top left corners show entire views of the embryos. (F’, G’) DIC images of F and G. White arrows indicate the nuclei. F and F’, and G and G’ are on the same focal planes, indicating that faint signals in the nuclei in G are not due to being out of focus for the nuclear level. The digits in the bottom right corner show the proportions of embryos with nuclear Pem signals. Scale bar, 50 μm. The proportions are also shown in H. ** P<0.01 (chi-square test).

In the process of searching for other factors in addition to Pem that regulate transcriptional repression in the embryonic germline, we found that Popk-1 knockdown by MO injection resulted in ectopic *FoxD*.*a* expression in B5.2 blastomeres, the germline cells of the 16-cell stage embryos (Fig 2A, 2B, arrows, 2E). This result indicates that Popk-1 is necessary to repress germline transcription at the same stage as Pem does. In the previous study, Popk-1 knockdown resulted in decrease of the CAB size as well as the amount of *postplasmic/PEM* RNAs localized to CAB [37], where translation of the localized mRNAs takes place [49]. Therefore, we hypothesized that Popk-1 represses germline gene expression indirectly via proper formation of CAB and production of proteins from the localized mRNAs.

To test our hypothesis, we fixed embryos at the 16-cell stage, which have been injected with *Popk-1* MO, and immunohistochemically stained Pem protein. Popk-1 knockdown resulted in a reduction of the Pem protein level in the germline nuclei (Fig 2G, white arrows), when compared with that of the control embryos (Fig 2F, white arrows). Further, consistent with the previous report [37], the CAB size seemed to become smaller in Popk-1 knockdown embryos as less localized Pem protein to this structure was observed (Fig 2F, 2G, white arrowheads). It has been shown that it is Pem protein in the nucleus that represses germline gene expression [8]. Therefore, Popk-1 likely functions to maintain the nuclear Pem protein level required for transcriptional repression. To further investigate whether Popk-1 regulates germline transcription via Pem, we analyzed the effect of Pem overexpression on the ectopic *FoxD*.*a* expression caused by knockdown of Popk-1. Co-injection of *Popk-1* MO and *Pem* mRNA abolished the ectopic signal in the germline as well as signals in the somatic cells (Fig 2C–2E). Thus, Popk-1 regulates the repression of germline gene expression indirectly by maintaining Pem protein level.

### Initiation of zygotic gene expression in the germline

Germline cells are silent in transcription at a certain stage of development; however, they eventually start gene expression to differentiate into gametes. We next sought to understand how the de-repression of transcription in the germline is regulated in *Halocynthia* embryos. In previous research, *Pem* mRNA and protein levels have been shown to be reduced as embryogenesis proceeds [47]. We hypothesized that decreasing the Pem level may trigger de-repression of the germline gene expression.

To address this question, we first explored the EST database of *Halocynthia*, MAGEST [41], for genes that show zygotic expression in the embryonic germline. It is known that 20 genes are registered as those expressed in the germline of the tailbud embryo [41]. Of these, a gene called *Clone 45* or *ADP/ATP translocase* shows strong expression in the germline, and is therefore selected as a candidate marker gene for zygotically expressed germline genes.

To detect the onset of zygotic expression of this gene, we performed WISH for *ADP/ATP translocase*. *ADP/ATP translocase* was expressed in the germline as reported previously [41] as well as mesenchyme cells at the early neurula stage and later (S2 Fig). However, germline signals at the earlier stages could not be detected due to the high background of staining possibly caused by maternal transcripts. Therefore, we could not determine the timing of when zygotic *ADP/ATP translocase* expression starts (S2 Fig).

**Fig 3:**
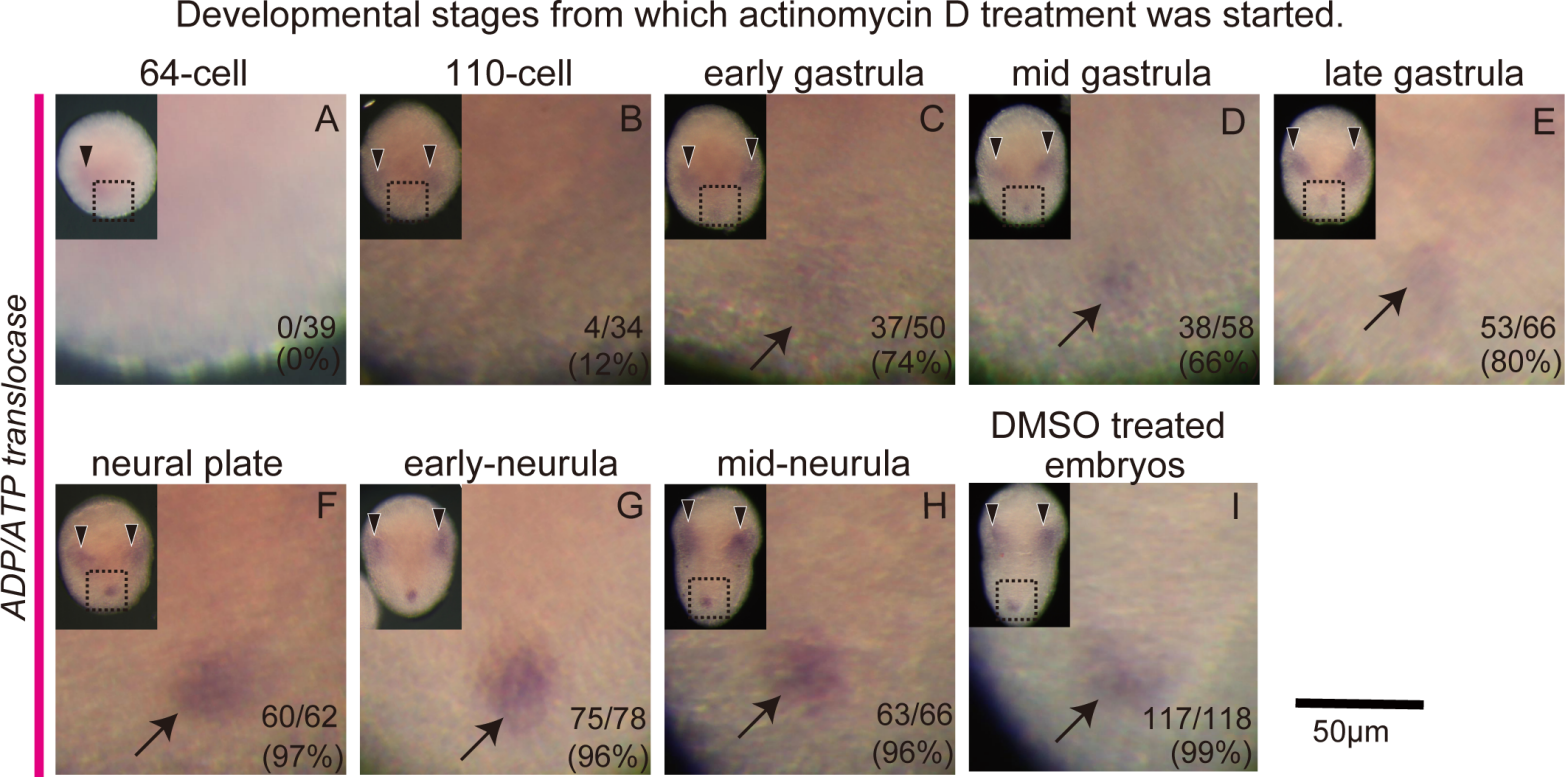
Zygotic gene expression of *ADP/ATP translocase* in the germline. (A-I) WISH images probed for *ADP/ATP translocase*. Ventral views. Anterior is to the top. Developmental stages above each image show the stages from which the actinomycin D treatment was started. Arrows indicate germline expression and arrowheads somatic, possibly mesenchymal, expression. Smaller images in the top left corners are entire views of the embryos. The digits in the bottom right corners indicate the proportion of embryos with germline expression. Scale bar, 50μm.

To overcome this problem, we treated embryos with a transcriptional inhibitor, actinomycin D. We started the inhibitor treatment at various stages ranging from the 64-cell to the mid neurula stage, and fixed the embryos for WISH at late neurula stage (Fig 3), when the background staining was decreased (S2 Fig).

In DMSO-treated control embryos (Fig 3I) and embryos that were treated with actinomycin D starting at and after the neural plate stage (Fig 3F–3H), clear *ADP/ATP translocase* signals in the germline were detected in more than 95% of cases (control embryos n = 117/118 (99%); Fig 3F–3I). However, fewer embryos showed gene expression in the germline (Fig 3A–3E) the earlier the treatment started, and in the embryos that were treated from the 110-cell stage signal was observed in only about 12% of the embryos (Fig 3B). These results suggest that the *ADP/ATP translocase* signal detected in the germline is indeed derived from zygotic expression, and that its zygotic expression starts around the onset of gastrulation.

### Decreased Pem protein level triggers zygotic germline gene expression

**Fig 4:**
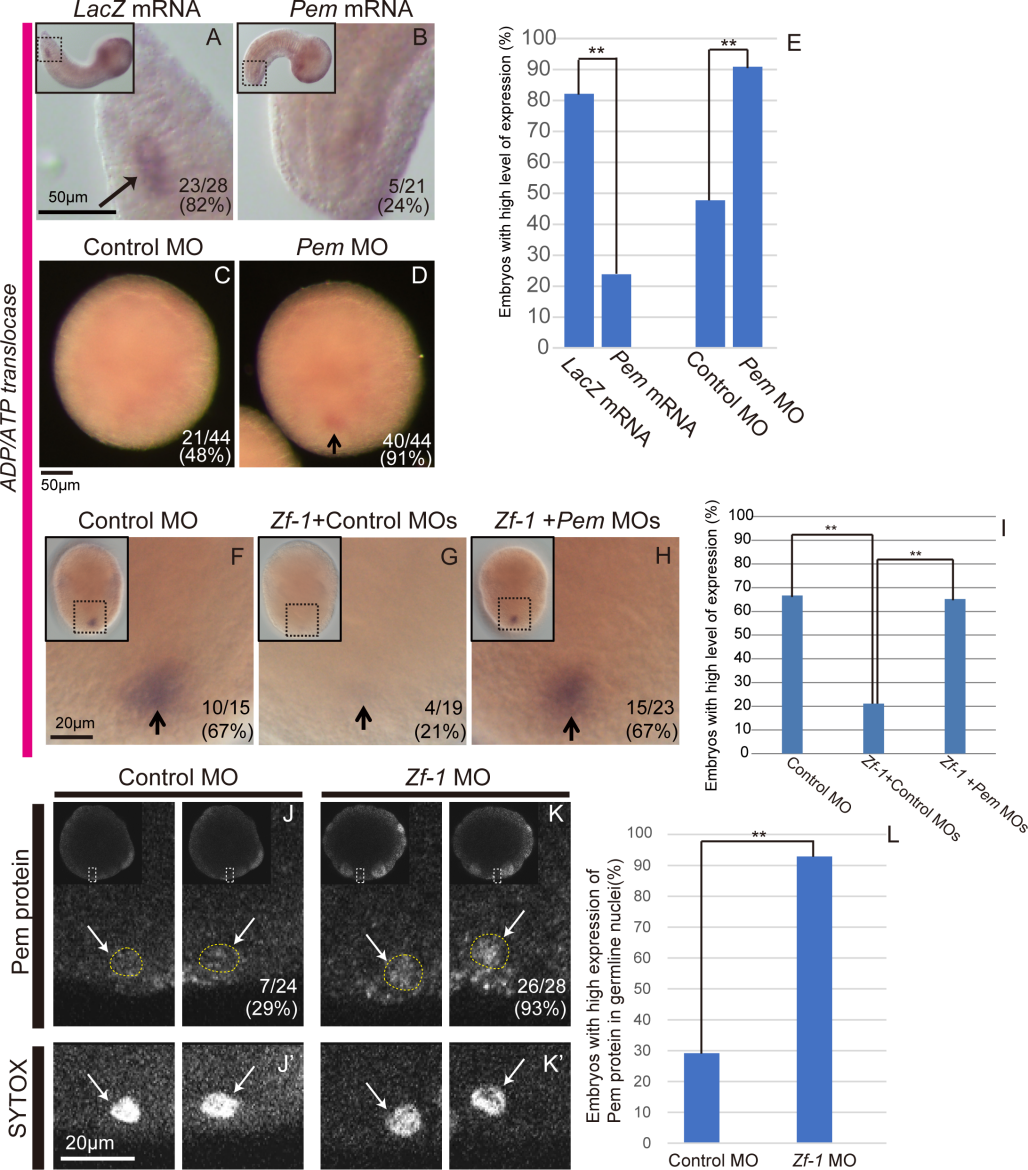
Regulation of zygotic expression of *ADP/ATP translocase* by Pem and Zf-1. (A, B) WISH images for *ADP/ATP translocase*. (A) 50 pg of *LacZ* mRNA- and (B) 50 pg of *Pem* mRNA-injected embryos. Arrows indicate *ADP/ATP translocase* signal in the germline. (C, D) Images of actinomycin D-treated embryos stained for *ADP/ATP translocase* expression. Vegetal views. Anterior is to the top. (C) 100 pg of control MO- and (D) 100 pg of *Pem* MO-injected embryos. Black arrow in D indicates the *ADP/ATP translocase* signal in the germline. The digits in the bottom right corner show the proportion of embryos with germline expression. (E) Proportion of embryos with *ADP/ATP translocase* expression in the germline. ** P<0.01 for A-D (chi-square test). (F) 300 pg of control MO-, (G) 200 pg of *Zf-1* and 100 pg of control MOs-, and (H) 200 pg of *Zf-1* and 100 pg of *Pem* MOs-injected embryos. Arrows in F-H indicate the *ADP/ATP translocase* signal in the germline. Scale bars for A-D, 50 μm; that for F-H, 20 μm. (I) Proportion of embryos in which the *ADP/ATP translocase* expression was observed in the germline. ** P<0.01 (chi-square test). (J, K) B7.6 cells of the 64-cell stage embryos stained with anti-Pem antibody. 100–350 pg of control–or 100–350 pg of *Zf-1* MO were injected in this experiment. (J) 100 pg of control- and (K) 100 pg of *Zf-1* MO-injected embryos. Arrows indicate the Pem protein signals in the nuclei. The digits in the bottom right corner show the proportion of embryos with the high expression level of nuclear Pem signals. These panels are single focal planes, where the Pem protein signals were strongest in the Z-series through the nuclei. J and J’, and K and K’ are on the same focal planes. Scale bar, 20 μm. (A, B, F-H, J, K) Smaller panels in the top left corners show entire embryos. (L) Proportion of embryos showing higher nuclear signals. **P<0.01 (chi-square test).

After *ADP/ATP translocase*, a zygotically expressed germline gene, was identified we next examined whether the decrease of Pem protein level de-represses the expression of *ADP/ATP translocase*. We first attempted to slow down the decrease of Pem by overexpressing Pem in the germline. We injected the germline B5.2 blastomeres on both sides with *Pem* mRNA at the 16-cell stage and cultured the embryos until the mid-tailbud stage. We found that the *ADP/ATP translocase* expression was reduced compared with that in the *LacZ* mRNA-injected control embryos (Fig 4A, 4B and 4E). These results suggest that a decrease of Pem protein level during embryogenesis is necessary for zygotic *ADP/ATP translocase* expression.

We next investigated whether *ADP/ATP translocase* expression starts earlier by injecting *Pem* MO and facilitating precocious reduction of the Pem protein level. We injected eggs with either control or *Pem* MO, and the embryos were then treated with actinomycin D from the 110-cell stage and fixed at the late neurula stage. Germline expression was observed in nearly half of the embryos injected with control MO. In contrast, germline expression was observed in almost all Pem knockdown embryos (Fig 4C–4E). These results suggest that Pem knockdown shifted the onset of zygotic germline gene expression earlier and that decreasing Pem protein level resulted in precocious zygotic *ADP/ATP translocase* expression. These results indicate that Pem suppresses somatic gene expression in the germline [8] and its reduction regulates the timing of zygotic germline gene expression.

### Zf-1 promotes reduction of Pem protein level

Finally, we attempted to identify a factor(s) that also affect the timing of zygotic *ADP/ATP translocase* expression. We injected fertilized eggs with several kinds of MOs, and cultured the injected embryos in the presence of actinomycin D from the neural plate stage to the late neurula stage when we fixed them for *ADP/ATP translocase* WISH. The actinomycin D treatment made it possible to mainly detect the initial stage of zygotic *ADP/ATP translocase* expression in the germline. We found that the *ADP/ATP translocase* expression was significantly lower than that in control embryos when we injected them with MO against Zf-1 (Fig 4F, 4G and 4I). The reduction of *ADP/ATP translocase* expression by Zf-1 knockdown was partially rescued by co-injection of *Zf-1* mRNA (S3 Fig). Although the current condition in the rescue experiment did not give a high rate of successful rescue, this result suggests that the effect of the MO used here is specific. These results suggest that Zf-1 is required for the onset of zygotic expression of *ADP/ATP translocase* in the germline. *Zf-1* is a member of the *postplasmic/PEM* RNAs and encodes C3H-type zinc finger protein [46, 50].

We next examined whether Zf-1 regulates *ADP/ATP translocase* expression via controlling the Pem protein level. We found that co-injection of *Pem* MO together with *Zf-1* MO restored *ADP/ATP translocase* expression in the germline (Fig 4H and 4I), indicating that Zf-1 promotes *ADP/ATP translocase* expression through negatively regulating Pem. In accordance with the above result, Zf-1 knockdown resulted in the increase in the level of Pem protein in the germline nuclei of the 64-cell stage embryo (Fig 4J–4L). Pem protein signal was only slightly detected in the germline nuclei (Fig 4J, arrows) at the 64-cell stage; however, it was observed at a higher level in the nuclei (Fig 4K, arrows) in the Zf-1 knockdown embryos. These results support our hypothesis that Zf-1 upregulates zygotic *ADP/ATP translocase* expression by reducing the Pem protein level.

## Discussion

### Germ plasm formation and transcriptional repression

The present study suggests that Popk-1 contributes to germline transcriptional repression indirectly via regulating proper CAB formation. Since CAB is the site where *postplasmic/PEM* RNAs are localized [29, 31, 32, 50] and presumably where their translation takes place [48], it is likely that Popk-1 regulates the function of other *postplasmic/PEM* RNAs including *Pem* by controlling the amount of the localized mRNAs and of the translated proteins at CAB necessary for their proper function. Consistently, Popk-1 has been shown to act upstream of *Macho-1* mRNA, another *postplasmic/PEM* RNAs member [51], and regulate the formation of posterior tissues such as muscle and mesenchyme [51, 52].

Localized maternal factors that assemble the germ plasm have been identified in other animals, such as Oskar of *D*. *melanogaster* [53–56], PGLs of *C*. *elegans* [57], Bucky ball of zebrafish [58] and Xpat of *X*. *laevis* [59]. These factors function to assemble other maternal mRNAs and proteins in the germ plasm, and are essential for PGC formation. In this sense, Popk-1 could be considered to be among these factors since Popk-1 assembles sufficient amount of *Pem* mRNA for its function to the ascidian germ plasm (CAB) and its knockdown resulted in ectopic somatic gene expression in PGC (Fig 2). However, whereas those four factors in the non-ascidian species mentioned above are all taxon-specific [35, 36, 57, 59], Popk-1 is an ascidian orthologue to SAD kinases, which play important roles in axonal development in mouse and *C*. *elegans* [60, 61]. It would be interesting to examine whether Popk-1/SAD functions in germline formation is conserved in other animal species.

### De-repression of gene expression after the disappearance of Pem

The attenuation of the Pem protein level during embryogenesis is necessary and sufficient for the onset of zygotic gene expression in the germline. A previous antibody staining against Pem revealed that the Pem protein level gradually decreases from the 32- to 110-cell stages [47]. This is consistent with our finding that the expression of a germline zygotic gene *ADP/ATP translocase* starts at around the 110-cell and early gastrula stages (Fig 3). In *C*. *elegans* embryos, zygotic gene expression such as that involved in gamete differentiation is activated in Z2 and Z3 germline cells at around the 100-cell stage when PIE-1 protein disappears [3, 62]. Importantly, somatic gene expression should remain silent even after the RNA polymerase II-dependent transcriptional repression by maternal factors such as Pem and PIE-1 is lifted and zygotic gene expression starts in the germline. It is not known in ascidian embryos how germline and somatic genes are differently regulated at this stage, however, chromatin-based epigenetic silencing mechanisms are suggested to follow the repression mechanisms by Pgc in *D*. *melanogaster* and PIE-1 in *C*. *elegans* [2, 3]. Therefore, a similar mechanism could also take over after Pem in ascidian embryos.

### Regulation of somatic and germline gene expression by Pem

One of the interesting findings from the current studies is that attenuation of Pem protein level is essential for zygotic germline gene expression. Previously, Pem was shown to repress expression of every somatic gene tested such as *FoxA*, and *FoxD*.*a* in the germline at the 8- and 16-cell stages and a subset of them at the 32-cell stage in *H*. *roretzi* embryos [8]. However, *FoxA* and *FoxD*.*a* were no longer ectopically expressed in the germline upon Pem knockdown at the 32-cell stage [8], suggesting that the Pem-dependent mechanism may be gradually replaced by others, as discussed above, as early as the 32-cell stage. In contrast, Pem appears to repress the onset of zygotic gene expression in the germline until the 110-cell and early gastrula stages.

Therefore, there might be a time difference between when zygotic germline gene expression starts due to the decrease in the Pem protein level (110/early gastrula stage) and when the effect of Pem on the repression of somatic gene expression in the germline attenuates (32-cell stage). The earlier replacement of the repression mechanisms around the 32-cell stage may ensure that somatic genes will never be expressed in the germline when the Pem protein level is gradually decreased nearing the onset of zygotic gene expression. Somatic gene expression may be more susceptible to the decrease in the Pem protein level because somatic genes such as *FoxA* become expressed in somatic daughter cells (B5.1) as soon as they are separated from the germline daughter (B5.2) and from the source of Pem protein (CAB) after the cell division of the mother germline cells (B4.1) [8, 38].

### Decrease in the Pem protein level by mediated Zf-1

Our results suggest that Zf-1 decreases the Pem protein level and consequently regulates the timing of zygotic gene expression. How does Zf-1 reduce the Pem protein level? Zf-1 encodes a C3H-type zinc finger protein predicted to bind RNA [46, 50].

Therefore Zf-1 could decrease the Pem protein level by translation inhibition or the mRNA degradation via its binding to *Pem* mRNA. We prefer the former possibility because *Pem* mRNA was still detected, while Pem protein was absent in the germline of the tailbud stage embryo (S4 Fig). Zf-1 protein is probably translated from its maternal mRNA and gradually accumulated during embryogenesis to the extent sufficient enough to repress the translation of maternally supplied *Pem* mRNA during the cleavage stages [47]. Translational repression by RNA binding proteins is known to play important roles in germline development [4, 35, 63]. For example, an RNA binding protein Bruno in *D*. *melanogaster* contributes to the proper localization of Oskar protein via translational repression of *Oskar* mRNA [56, 64].

### Overview

**Fig 5:**
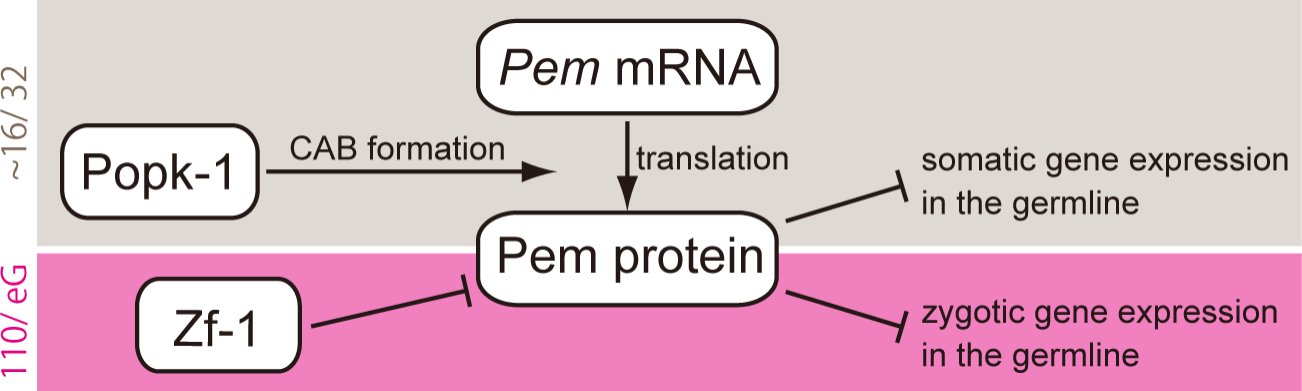
A model for germline transcriptional regulation by *postplasmic/PEM* RNAs.

In addition to Pem, which has been shown to regulate germline development through transcriptional regulation in ascidian embryos [8], two other members of the *postplasmic/PEM* RNAs, namely Popk-1 and Zf-1, are involved in positive and negative post-transcriptional regulation of Pem during ascidian germline development, respectively (Fig 5). We propose that the regulation of the Pem protein level is crucial for proper transcriptional control in the germline, both of the repression of somatic gene expression and the onset of zygotic gene expression.

## Supporting information

S1 Fig Influence of higher concentration of Popk-1 MO. (A, B) WISH images probed for *FoxD*.*a*. (A) 1500 pg of control MO-, (B) 1500 pg of *Popk-1* MO-injected embryos fixed at the 16-cell stage. Vegetal views. Anterior is to the top. Arrowheads indicate germline B5.2 cells. The digits in the bottom right corner indicate the proportions of embryos with ectopic *FoxD*.*a* expression in the germline.
(TIF)

S2 Fig *ADP/ATP translocase* expression during *H*. *roretzi* embryogenesis. Embryos stained for *ADP/ATP translocase* expression. Stages at which the expression was detected are indicated above the images. Arrows indicate the *ADP/ATP translocase* expression in the germline. The digits in the bottom right corner indicate proportions of the embryos in which the *ADP/ATP translocase* signals were visible in the germline. Prior to the neurula stage, the expression could not be detected because of high background of staining due to the maternal mRNA. n.d. is not determined. Upper left panels show the entire view of the embryos. Dotted squares represent the cropped areas. Arrowheads in D-H indicate the signals in somatic, possibly mesenchymal, cells. Anterior is at the top. Embryos in A-F are vegetal views, and those in G, H are lateral views. Scale bar, 50 μm.
(TIF)

S3 Fig Specificity of Zf-1 MO. (A-C) WISH images for *ADP/ATP translocase*. (A) Control un-injected, (B) 100 pg of *Zf-1* MO and 200 pg of *LacZ* mRNA- and (C) 100 pg of *Zf-1* MO and 200 pg of *Zf-1* mRNA-injected embryos. Ventral views. Arrows indicate *ADP/ATP translocase* signal in the germline. The digits in the bottom right corner indicate proportions of the embryos with *ADP/ATP translocase* expression in the germline. All these embryos were treated with actinomycin D from the neural plate stage and fixed for WISH at the late neurula stage. Scale bar, 50 μm. (D) Proportion of embryos in which the *ADP/ATP translocase* expression was observed in the germline. ** P<0.01 (chi-square test).
(TIF)

S4 Fig Presence of *Pem* mRNA but not Pem protein in the tailbud embryo. (A) WISH for *Pem* mRNA. Black arrow indicates the *Pem* signal in the germline. White arrowhead indicates the same, but for an unknown reason it is spatially separated from that shown by the black arrow. This could be an equivalent of B8.11 blastomeres, the sister cell to the germline B8.12, identified in *C*. *robusta* [24]. The digit in the bottom right corner indicates the proportion of positive embryos. (B) Antibody staining with anti-Pem antibody. No signal was detectable. Scale bars, 50 μm. Smaller panels show the entire views of the tailbud embryos.
(TIF)
