# Supplementary figures and images for: Control of Pem protein level by localized maternal factors for transcriptional regulation in the germline of the ascidian, Halocynthia roretzi
Source: PLoS One. 2018 Apr 30;13(4):e0196500. doi: 10.1371/journal.pone.0196500 (PMC5927453; doi:10.1371/journal.pone.0196500)

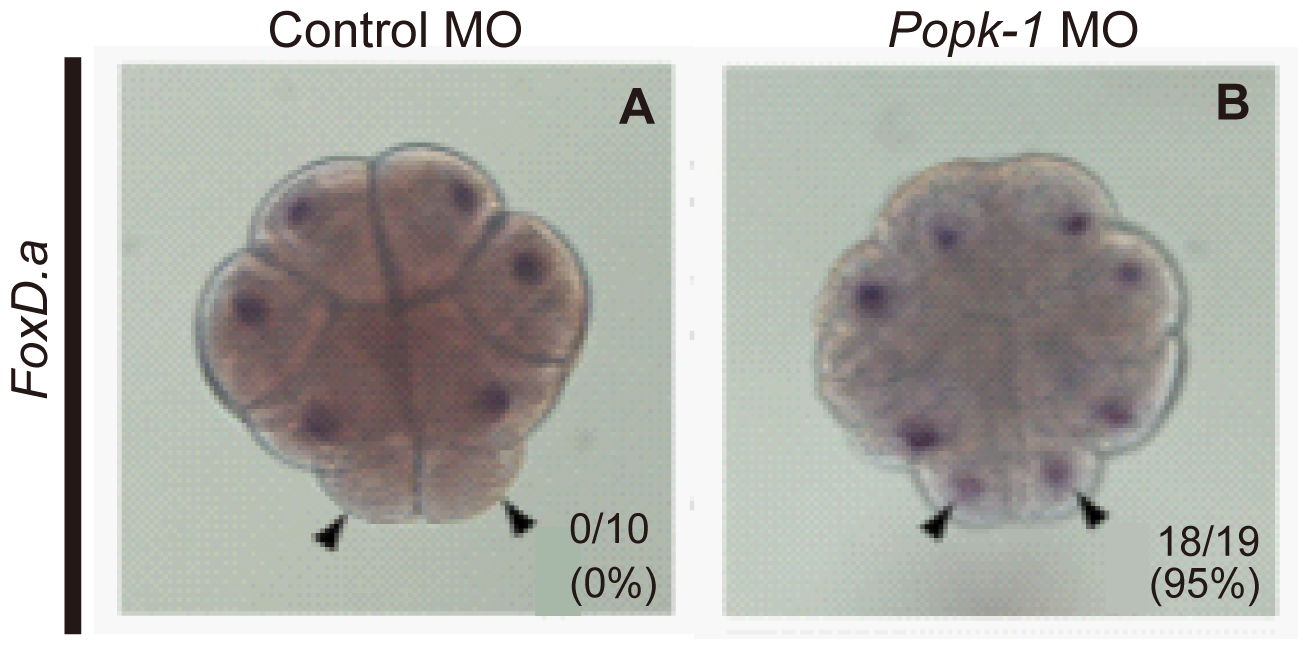

Supplement: S1 Fig — (A, B) WISH images probed for FoxD.a. (A) 1500 pg of control MO-, (B) 1500 pg of Popk-1 MO-injected embryos fixed at the 16-cell stage. Vegetal views. Anterior is to the top. Arrowheads indicate germline B5.2 cells. The digits in the bottom right corner indicate the proportions of embryos with ectopic FoxD.a expression in the germline. (TIF) [file pone.0196500.s001.tif]

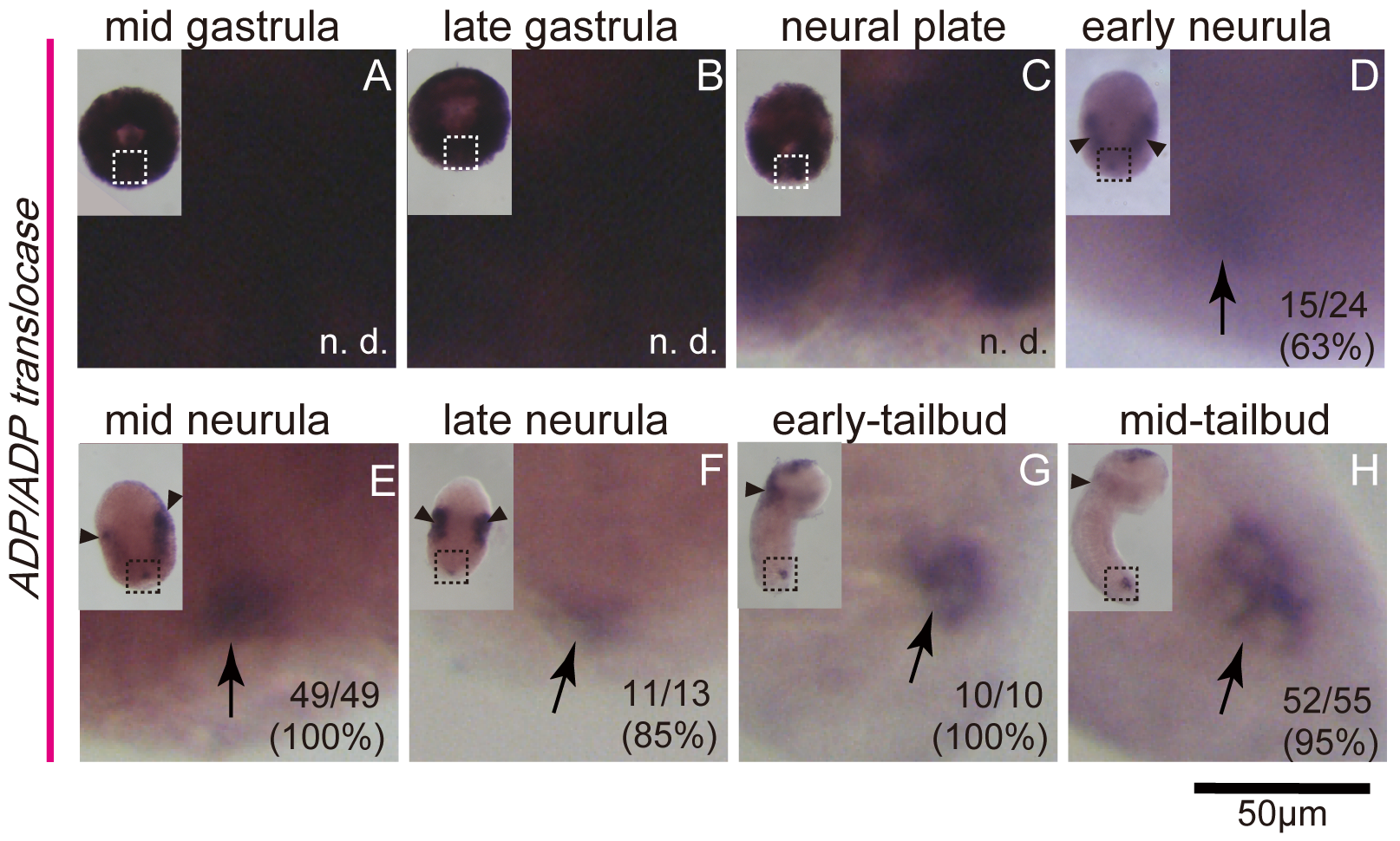

Supplement: S2 Fig — Embryos stained for ADP/ATP translocase expression. Stages at which the expression was detected are indicated above the images. Arrows indicate the ADP/ATP translocase expression in the germline. The digits in the bottom right corner indicate proportions of the embryos in which the ADP/ATP translocase signals were visible in the germline. Prior to the neurula stage, the expression could not be detected because of high background of staining due to the maternal mRNA. n.d. is not determined. Upper left panels show the entire view of the embryos. Dotted squares represent the cropped areas. Arrowheads in D-H indicate the signals in somatic, possibly mesenchymal, cells. Anterior is at the top. Embryos in A-F are vegetal views, and those in G, H are lateral views. Scale bar, 50 μm. (TIF) [file pone.0196500.s002.tif]

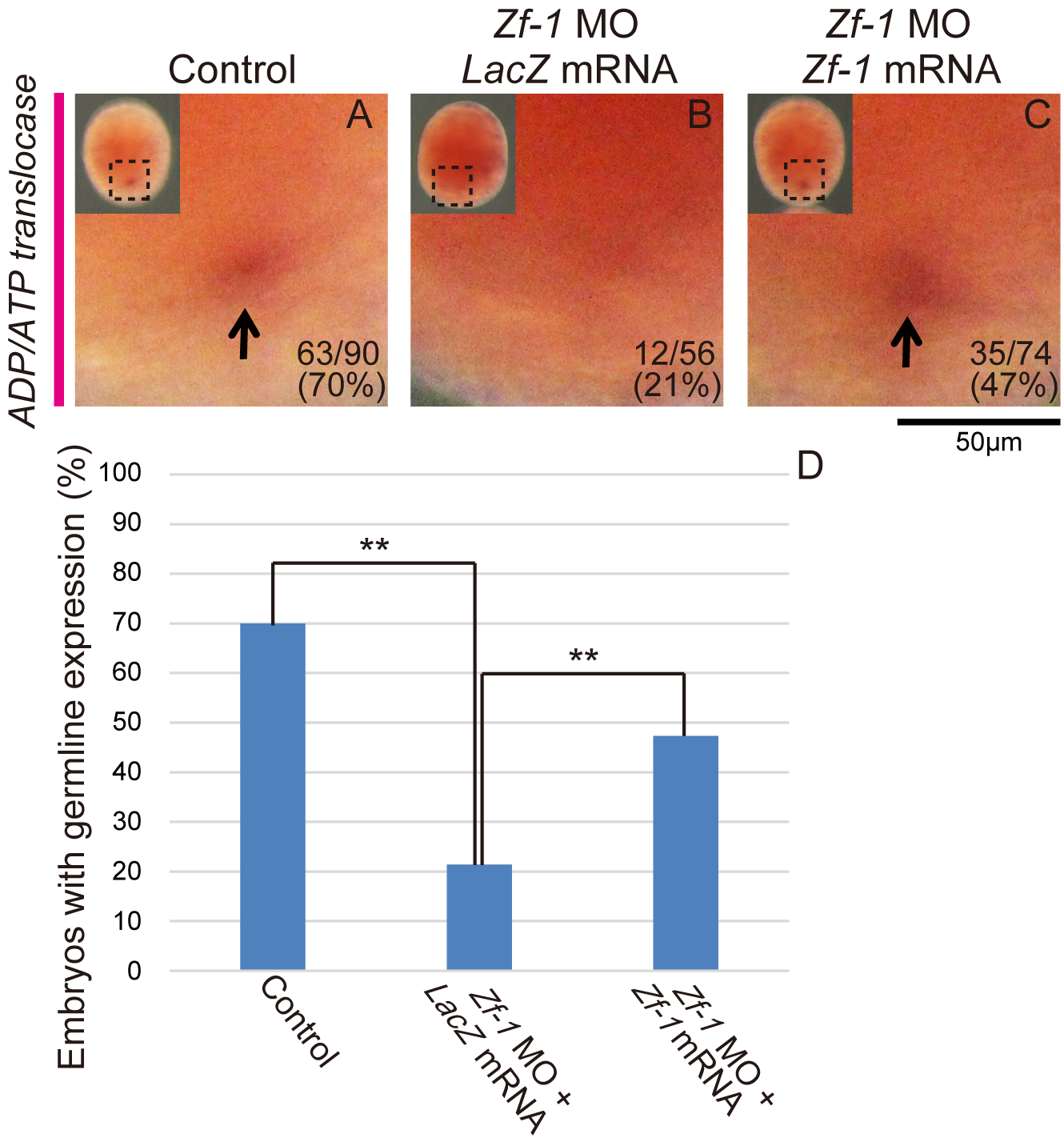

Supplement: S3 Fig — (A-C) WISH images for ADP/ATP translocase. (A) Control un-injected, (B) 100 pg of Zf-1 MO and 200 pg of LacZ mRNA- and (C) 100 pg of Zf-1 MO and 200 pg of Zf-1 mRNA-injected embryos. Ventral views. Arrows indicate ADP/ATP translocase signal in the germline. The digits in the bottom right corner indicate proportions of the embryos with ADP/ATP translocase expression in the germline. All these embryos were treated with actinomycin D from the neural plate stage and fixed for WISH at the late neurula stage. Scale bar, 50 μm. (D) Proportion of embryos in which the ADP/ATP translocase expression was observed in the germline. ** P<0.01 (chi-square test). (TIF) [file pone.0196500.s003.tif]

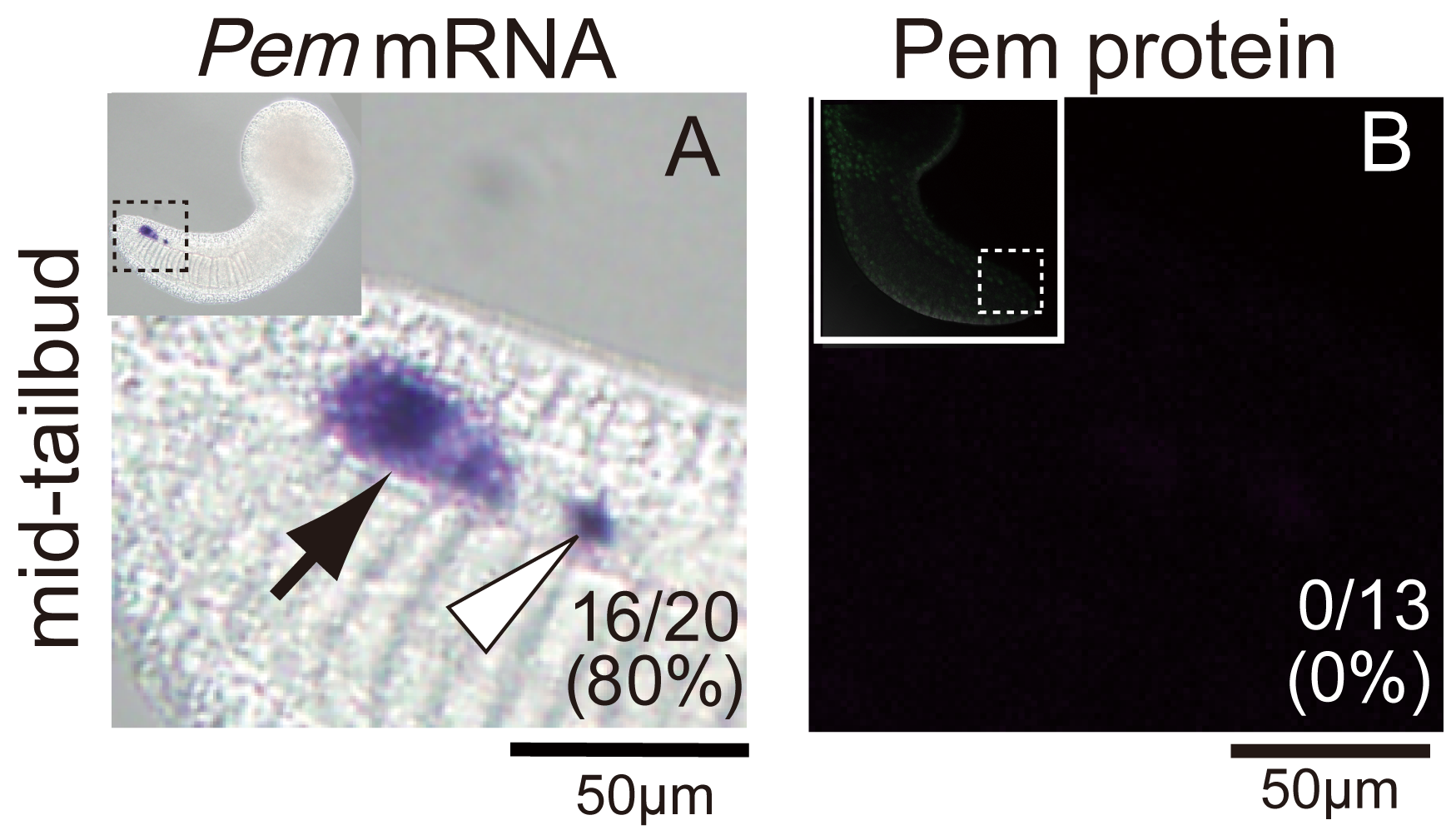

Supplement: S4 Fig — (A) WISH for Pem mRNA. Black arrow indicates the Pem signal in the germline. White arrowhead indicates the same, but for an unknown reason it is spatially separated from that shown by the black arrow. This could be an equivalent of B8.11 blastomeres, the sister cell to the germline B8.12, identified in C. robusta [24]. The digit in the bottom right corner indicates the proportion of positive embryos. (B) Antibody staining with anti-Pem antibody. No signal was detectable. Scale bars, 50 μm. Smaller panels show the entire views of the tailbud embryos. (TIF) [file pone.0196500.s004.tif]
